# Extremely high electrical conductance of microporous 3D graphene-like zeolite-templated carbon framework

**DOI:** 10.1038/s41598-017-11602-5

**Published:** 2017-09-13

**Authors:** Hyunsoo Lee, Kyoungsoo Kim, Seoung-Hun Kang, Yonghyun Kwon, Jong Hun Kim, Young-Kyun Kwon, Ryong Ryoo, Jeong Young Park

**Affiliations:** 1 Center for Nanomaterials and Chemical Reactions, Institute for Basic Science (IBS), Daejeon, 34141 South Korea; 2 Department of Physics and Research Institute for Basic Sciences, Kyung Hee University, Seoul, 02447 South Korea; 3 Korea Institute for Advanced Study, Seoul, 02455 South Korea; 4 Department of Chemistry, Korea Advanced Institute of Science and Technology (KAIST), Daejeon, 34141 South Korea; 5 Graduate School of EEWS, Korea Advanced Institute of Science and Technology (KAIST), Daejeon, 34141 South Korea; 6 Present Address: Department of Chemistry, Chonbuk National University, Jeonju, Jeollabuk-do 54896 South Korea; 7 Present Address: Department of Materials Science and Engineering, Yonsei University, Seoul, 03722 South Korea

## Abstract

We report the remarkably high electrical conductance of microporous 3D graphene-like carbons that were formed using lanthanum (La)-catalyzed synthesis in a Y zeolite (LaY) template investigated using conductive atomic force microscopy (C-AFM) and theoretical calculations. To uncover the relation between local electrical conductance and the microporous structures, we tuned the crystallographic ordering of LaY-templated carbon systems by changing the heating temperature. The structure of the LaY-templated carbon prepared at the higher temperature has graphene-like *sp* ^*2*^ hybridized bonds, which was confirmed using high-resolution transmission electron microscopy and X-ray diffraction measurements. C-AFM current–voltage spectroscopy revealed that the local current flow in the LaY-templated carbon depends on the quantity of C–C bonds within the narrow neck between the closed supercages (i.e. there are three types of carbon: carbon with heat treatment, carbon without heat treatment, and carbon synthesized at low temperature). The difference in electrical conductance on the LaY-templated carbon was also confirmed via theoretical computation using the Boltzmann transport theory and the deformation potential theory based on the density functional theory. These results suggest that the degree of order of the pores in the 3D zeolite-templated carbon structures is directly related to electrical conductance.

## Introduction

Ordered microporous carbons (i.e. pore size < 2 nm) synthesized using the nanocasting method with a zeolite as the sacrificial template are of scientific and technological interest owing to their regular and three-dimensional (3D) interconnected pore structure and high surface area greater than 2000 m^2^ g^−1^ ^1–10^. The synthesis requires a carbon deposition process within the zeolite micropores that uses small hydrocarbon molecules (e.g. acetylene, ethylene, propylene)^11^. This process is often troublesome because pyrolytic carbonization of the hydrocarbons can also occur outside the zeolite template. Carbon that forms on the external surfaces of the zeolite hinders further diffusion of the hydrocarbon molecules into the micropores, and consequently causes insufficient formation of the carbon framework to replicate the zeolite pore structure. To mitigate this problem, many researchers have developed carbon deposition techniques, including a two-step carbon infiltration method involving the impregnation of liquid furfuryl alcohol, chemical vapor deposition (CVD) of propylene^2, 3, 12^, a CVD process at very low pressure, and a pulsed CVD method^5, 11^. Very recently, we reported a facile method to selectively form a carbon structure inside zeolite micropores. Certain cations (e.g. La^3+^, Y^3+^, Ca^2+^) catalytically affect carbon formation in the zeolite pores at sub-nanometer scale through *d–π* coordination bonding with small olefinic hydrocarbons. That is, using a zeolite containing these cations, the carbon structure can be formed at a lower temperature where pyrolytic carbonization of the hydrocarbon normally does not occur. In this way, a 3D graphene-like *sp* ^2^ carbon framework was formed only inside the zeolite pores without carbon deposition on the external zeolite surfaces^13, 14^.

Zeolite-templated carbons (ZTCs) are now considered to be highly promising as an electrode material for lithium batteries, supercapacitors, and fuel cells. In battery and supercapacitor applications, ZTC electrodes can exhibit high specific capacitance even under high current density conditions because of the 3D connected and ordered pore network, which promotes the fast transport of electrolyte ions. In fuel cell applications, the novel structure of ZTC is highly effective for loading a large amount of Pt catalyst with high dispersion, leading to high activity for the electrocatalytic oxygen reduction reaction. Distinct from the structural characteristics of ZTCs, electrical conductance is another critical physical property for high performance in such electrical applications. The ability to conduct an electric current influences the internal resistance of the devices.

The electrical properties of ordered microporous carbon formed using a zeolite Y template were previously investigated. T. Kyotani *et al*. reported that for a porous carbon formed using a zeolite Y with the CVD process at high reaction temperature (i.e. about 800 °C), the electronic conductivity is lower than that of carbon from bulk polyacrylonitrile because a graphitized structure with high conductivity is not formed because of the spatial limitation in the zeolite Y channels^12^. In a further study, K. Nueangnoraj *et al*. reported the formation of a crosslinked fullerene-like framework that is close to carbon Schwarzite (i.e. a 3D graphene-based framework) using a zeolite Y replica with heat treatment at 900 °C after the CVD process. They mentioned that the ordered framework is structurally diverse (i.e. is both semiconductive with a bandgap and metallic with zero bandgap)^11^. To better understand and develop the electrochemical performance of ZTCs, investigation of local electrical conductance is very important.

Conductive atomic force microscopy (C-AFM) is a technique that shows the local electrical properties of electrically heterogeneous surfaces at nanometer scale because of the precise and stable electrical contact between the sharp AFM tip and the surface^15^. C-AFM has been widely used to reveal the local electrical conductance of various nanostructures, including nanodots^16^, nanorods^17^, tetrapods^18^, and nanodumbells^19^. In a recent report, we showed the synthesis of highly ordered microporous carbons using zeolites functionalized by carbonization catalysts (e.g. La^3+^, Y^3+^ or Ca^2+^) and their electrical conductance measured using C-AFM^13^. The carbons obtained from faujasite (FAU) zeolite templates exhibited electrical conductance two orders of magnitude greater than that of amorphous mesoporous carbon, which is explained by the graphene-like framework of the ZTC consisting of *sp* ^2^-bonded carbons. In this study, we shed further light on the electrical properties of the ZTC (i.e. lanthanum-catalyzed synthesis in a FAU zeolite template carbon) by means of different combinations of synthesis, measurement, and theoretical calculation. The crystallinity of the ZTC was controlled by changing the heating conditions, which produced several model systems for studying their electrical properties. Variation of the conductance, dependent upon the pore ordering and topology of the ZTC, is systematically investigated by using samples with tailored microporous structures (e.g. carbon with and without heat treatment, or synthesized at a lower temperature). We compare the electrical conductance of ZTCs in this study to theoretical values that are calculated using a computational model structure that utilizes the Boltzmann transport theory and deformation potential theory based on density functional theory (DFT).

## Results

### LaY zeolite-templated carbon

**Figure 1:**
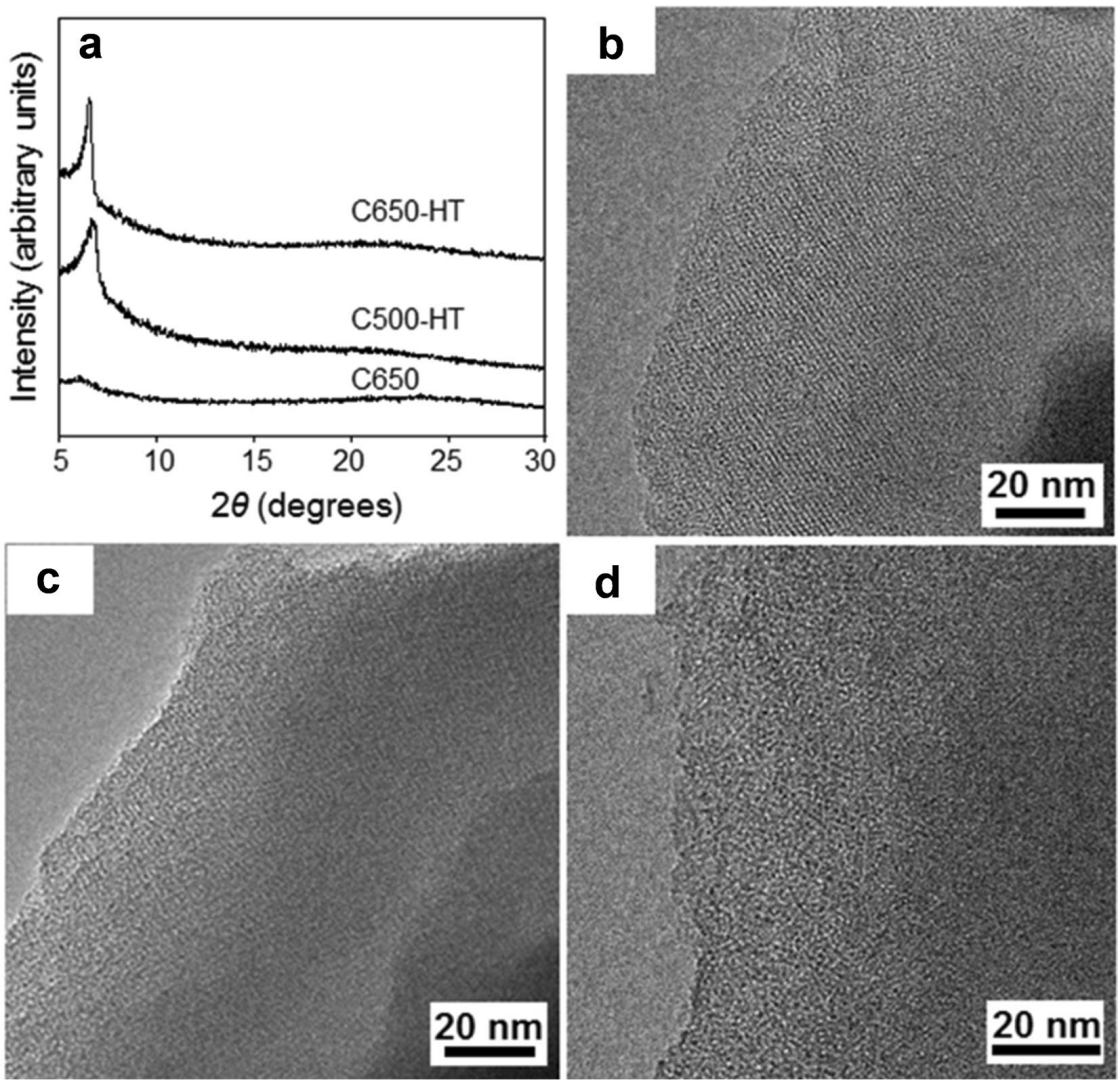
Structure of the LaY zeolite-templated carbon. (**a**) XRD patterns and (**b**–**d**) TEM images of the carbon samples obtained using LaY zeolite as the template under different synthesis conditions: (**b**) C650-HT, (**c**) C500-HT, and (**d**) C650.

Three types of carbon materials were prepared through carbonization of ethylene within a La^3+^-exchanged Y (LaY) zeolite. For two carbon samples (C500-HT and C650-HT), carbon deposition was performed at 500 and 650 °C, respectively, followed by thermal treatment at 900 °C under dry N_2_ flow. The other sample (C650) was synthesized using ethylene CVD at 650 °C without the subsequent thermal treatment. Figure 1 shows X-ray diffraction (XRD) patterns and transmission electron microscopy (TEM) images for the final carbon products after template removal. Both the C500-HT and C650-HT samples exhibit a well-resolved XRD peak at 2*θ* = 6.4° (Fig. 1a). This XRD peak position is almost the same as that of the (111) diffraction of the LaY zeolite. Accordingly, the XRD patterns of C650-HT and C500-HT indicate that the zeolite pore structure is successfully replicated by the carbon framework. Faithful carbon replication is supported by TEM images (Fig. 1b,c), showing a highly ordered microporous structure with lattice fringes of 1.4 nm. The fringe spacing is consistent with the XRD peak at 2*θ* = 6.4°. Previous reports in the literature show that heat treatment is required to densify the deposited carbon framework in the zeolite and to improve pore regularity^20–22^. On the other hand, the C650 sample obtained without thermal treatment exhibits a broad and weak XRD peak at around 2*θ* = 6.0° (Fig. 1a) and poorly-ordered pore arrays in the TEM image (Fig. 1d), which are probably caused by insufficient connectivity within the carbon frameworks without thermal treatment to retain the ordered porous structure after removing the template^1–3, 5, 12^. It is worth mentioning that none of the carbon samples have dense graphitic carbon layers outside the microporous carbon domains. The inhibition of carbon deposition on the external surfaces of the zeolite template can be attributed to the lower carbonization temperature^13^.

We investigated the carbon bonding in the samples using solid-state ^13^C magic-angle spinning NMR spectroscopy (Supplementary Fig. 1) and elemental analysis (Supplementary Table 1) (See Supplementary Information). All the NMR spectra are almost the same, showing predominant *sp* ^2^ carbon peaks at chemical shifts of 123 and 130 ppm. These peaks can be assigned to a 6-membered carbon ring and a distorted carbon ring (e.g. a 5- or 7-membered ring), respectively^13^. Thus, all the carbon samples are mainly composed of *sp* ^2^-hybridized carbon bonds. However, they have different hydrogen atom content, as evaluated by elemental analysis. The C/H atomic ratio of C650-HT is 8.9, while that of C500-HT is 5.7. The hydrogen content indicates the presence of edge-plane sites or vacancy defects in the graphene-like frameworks. That is, carbon deposition at temperatures that are too low can lead to formation of many fractures or vacant holes in the carbon framework even if the overall pore structure is well-ordered. On the other hand, the C650 sample exhibits a C/H ratio of 3.2, which can be related to a lack of framework connectivity, as expected from poor pore ordering. In addition, the Raman spectra exhibited no significant difference in the intensity ratio of the D- and G-bands or the band broadness for the carbon samples obtained using the different synthesis conditions because zeolite-templated carbons normally show a very broad intrinsic D-band that is attributed to the presence of five- or seven-membered carbon rings in the curved carbon structure (Supplementary Fig. 2)^3, 13, 23, 24^.

### Electrical conductivity

**Figure 2:**
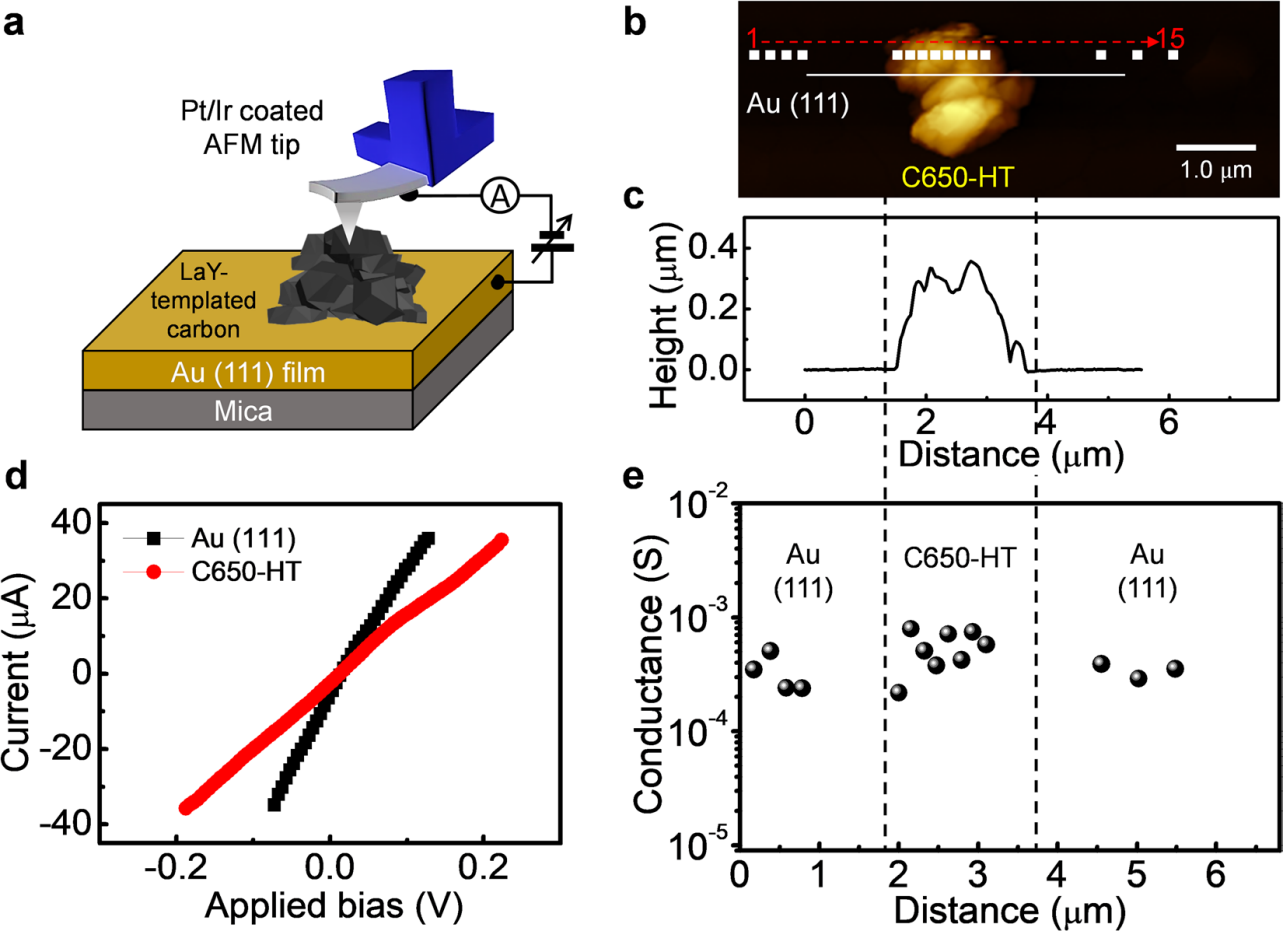
Electrical conductance of LaY zeolite-templated carbon with heat treatment. (**a**) Scheme for probing the electrical conductance of the LaY-templated carbon on Au (111)/mica using conductive AFM with a Pt/Ir metal-coated tip. (**b**) Topography (2.5 × 6.8 µm^2^) of C650-HT on Au (111). (**c**) Height line profile along the white solid line in (**b**). (**d**) I–V curves measured on C650-HT and Au (111) with a tip sweep bias of ±1 V in air. (**e**) Local electrical conductance of C650-HT and the Au (111) surface measured on the white dots in (**b**) at an applied load of 13 nN. The red dotted arrow with numbers in (**b**) indicates the direction and sequence of the I–V measurements.

To investigate the electrical conductance of the LaY-templated carbon, C-AFM was utilized because of its capability for local probing and for obtaining morphological and electrical properties under ambient conditions. Figure 2a shows the scheme for measuring the local electrical conductance of C650-HT on Au (111) (100 nm thickness) on a mica substrate using C-AFM with a Pt/Ir metal coated tip. The topography indicates randomly stacked LaY-templated carbons (bright parts) on the Au (111) surface after drop casting of carbon-dispersed isopropanol (Fig. 2b). The height and width of the stacked C650-HT are about 300 nm and 2 μm, respectively (Fig. 2c). Figure 2d shows representative current–voltage (I–V) curves measured on C650-HT with Au (111) as the reference. The Au (111) surface (3.5 × 10^−4^ Ω^−1^) shows a current flow only two times higher than that of C650-HT (1.7 × 10^−4^ Ω^−1^). Furthermore, we measured local I–V curves at the white square dots, moving in the direction of the red dotted arrow from left (first point) to right (fifteenth point), as marked in Fig. 2b. Positions 1–4 and 13–15 show the Au (111) surface and the other positions (i.e. 5–12) show C650-HT. The electrical conductance of C650-HT and the Au (111) surface seems indistinguishable because surface contamination of the Au (111) from the wet chemical processes and air can lead to varied and reduced local electrical conductance, as shown in Fig. 2e. Nevertheless, C650-HT consistently shows high electrical conductance.

**Figure 3:**
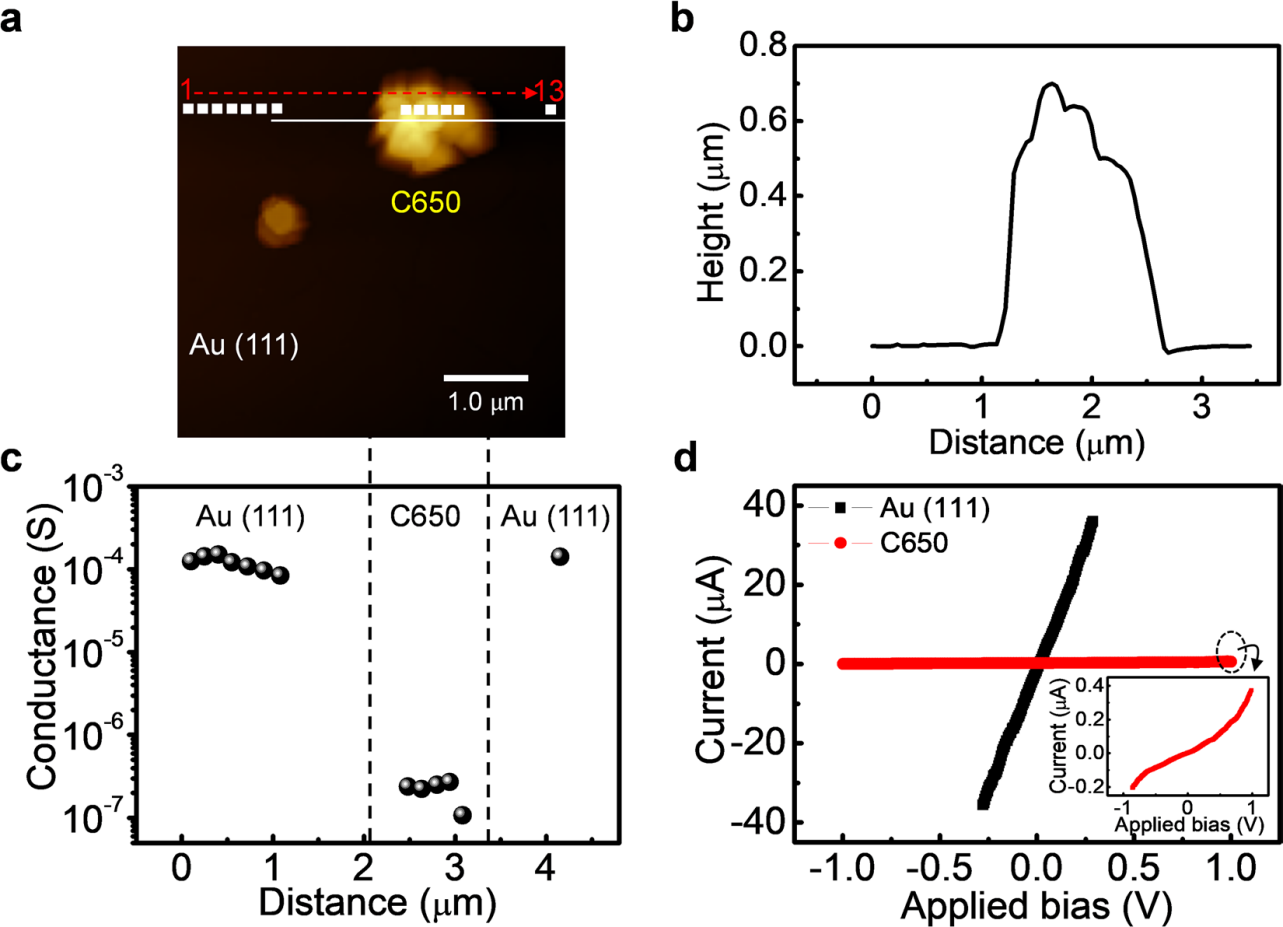
Electrical conductance of the LaY zeolite-templated carbon without heat treatment. (**a**) Topography (4.2 × 4.2 µm^2^) of C650 on Au (111). (**b**) Height line profile along the white solid line in (**a**). (**c**) Local electrical conductance of C650 and the Au (111) surface measured on the white dots in (**a**). (**d**) I–V curves measured on C650 and Au (111) at an applied load of 13 nN. The inset shows the magnified I–V curve of C650. The red dotted arrow with numbers in (**a**) indicates the direction and sequence of the I–V measurements.

Figure 3a show the topography of C650 on a Au (111) substrate. The morphology and dispersity of the LaY-templated carbon on the Au (111) surface are also confirmed by scanning electron microscope (SEM) imaging, as shown in Supplementary Fig. 3 (See Supplementary Information). The line profile in Fig. 3b shows a height and width of ~600 nm and ~1.5 μm, respectively. The representative I–V curves of C650 and Au (111) are shown in Fig. 3d. The inset in Fig. 3d shows the magnified I–V curve of C650. C650 (2.4 × 10^−7^ Ω^−1^) exhibits an electrical conductance three orders of magnitude lower than that of the Au (111) surface (1.2 × 10^−4^ Ω^−1^). Moreover, Fig. 3c shows the local electrical conductance of C650 and Au (111), which were measured at the white square dots, moving in the direction of the red dotted arrow from left to right, as marked in Fig. 3a. Positions 1–7 and 13 are on the Au (111) surface and positions 8–12 are on C650. The difference in electrical conductance is quite clear between the C650 and Au (111). The topography of C650 on Au (111) was taken before and after the I–V curve measurements to compare the morphology and position of the carbon (See Supplementary Fig. 4 in the Supplementary Information).

**Figure 4:**
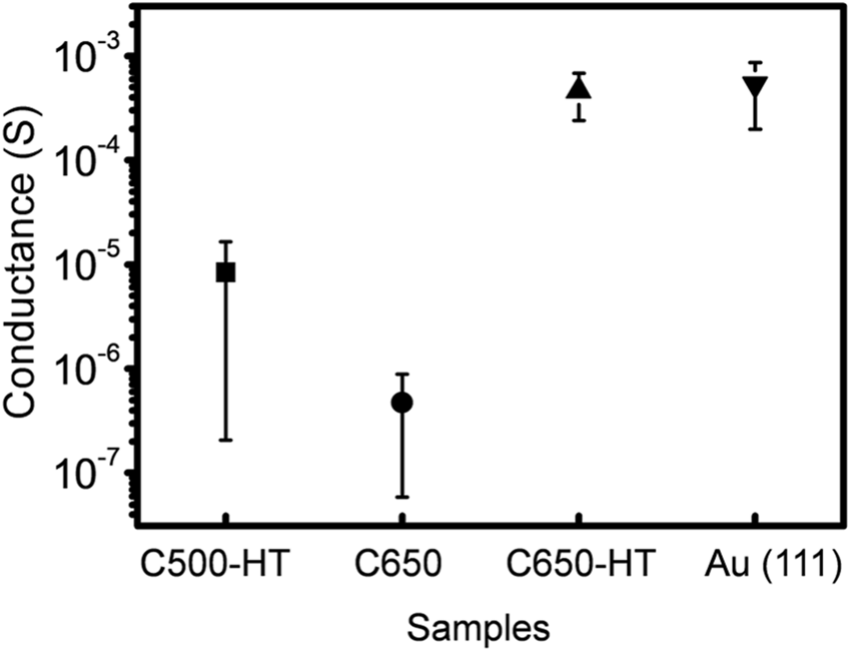
Electrical conductance of the LaY-templated carbon as a function of synthesis temperature and heat treatment. For the measurement, an applied load of 13 nN was used. The adhesion (effective load) is 15.6 nN (28.6 nN), 24.3 nN (37.3 nN), 18.2 nN (31.2 nN), and 50 nN (63 nN) for C650-HT, C650, C500-HT, and Au (111), respectively.

Figure 4 shows a comparison of the electrical conductance of the three different LaY-templated carbon samples and Au (111) as the reference. C650-HT clearly shows higher conductance than that of C650 and C500-HT, while C500-HT has a relatively high conductance range. The contact area between the probe and the surface can be obtained by using the Derjaguin–Muller–Toporov (DMT) model^25, 26^, given by *A* = π(*R* ^2/3^/*K* ^2/3^)(*L* + *L*_*c*_)^2/3^, where *R* is the curvature radius of the Pt/Ir-coated tip, *K* is the combined elastic modulus of the carbon (graphite) and the tip, *L* is the applied load, and *L*_*c*_ is the adhesion. The calculated contact area *A* is 6.6 ± 0.6 nm^2^ using *K* = 530 GPa^26, 27^; *R* = 50 nm ± 10; *L* = 13 nN; and *L*_*c*_ = 15.6 nN (C650-HT), 24.3 nN (C650), and 18.2 nN (C500-HT). The conductivity σ is 8.4 × 10^6^ S m^−1^ and 2.0 × 10^4^ S m^−1^ for C650-HT and C650, respectively.

## Discussion

The main question here is: “What is the origin of the difference in electrical conductance between C650-HT and C650?”. The most likely possibility is the degree of pore order in the carbon product due to the role of heat treatment. If a poorly ordered, synthesized carbon/zeolite composite is heat treated at about 900 °C, it becomes a carbon product with highly ordered pores. TEM data also show highly well-ordered carbon structures after heat treatment, as shown in Fig. 1. The lack of pore order is due to inadequate C–C bonds within the narrow necks between the closed supercages. Lack of pore order can also be caused by using a lower temperature during the carbon synthesis process even though heat treatment is performed. Thus, we also confirmed the electrical conductance of C500-HT, as shown in Supplementary Fig. 5 (See Supplementary Information). Additionally, X-ray photoelectron spectroscopy (XPS) was employed to analyze the chemical components of the three types of LaY-templated carbon (i.e. C650-HT, C650, and C500-HT), as shown in Supplementary Fig. 6 (See Supplementary Information). The C1s core-level spectrum revealed three major peaks, which are assigned to C–C/C–H bonds at 284.4 eV; the C–OH alcohol group at 286.1 eV; and the O = C–OH/O–C = O carbonate group at 288.6 eV. Comparing C650 and C500-HT, the portion of C–C/C–H was lower and C–OH was higher in C500-HT. The O1 s spectrum revealed two major peaks, which are assigned to C–O bonds at 531.7 eV and C=O bonds (i.e. of the alcohol group) at 532.9 eV.

**Figure 5:**
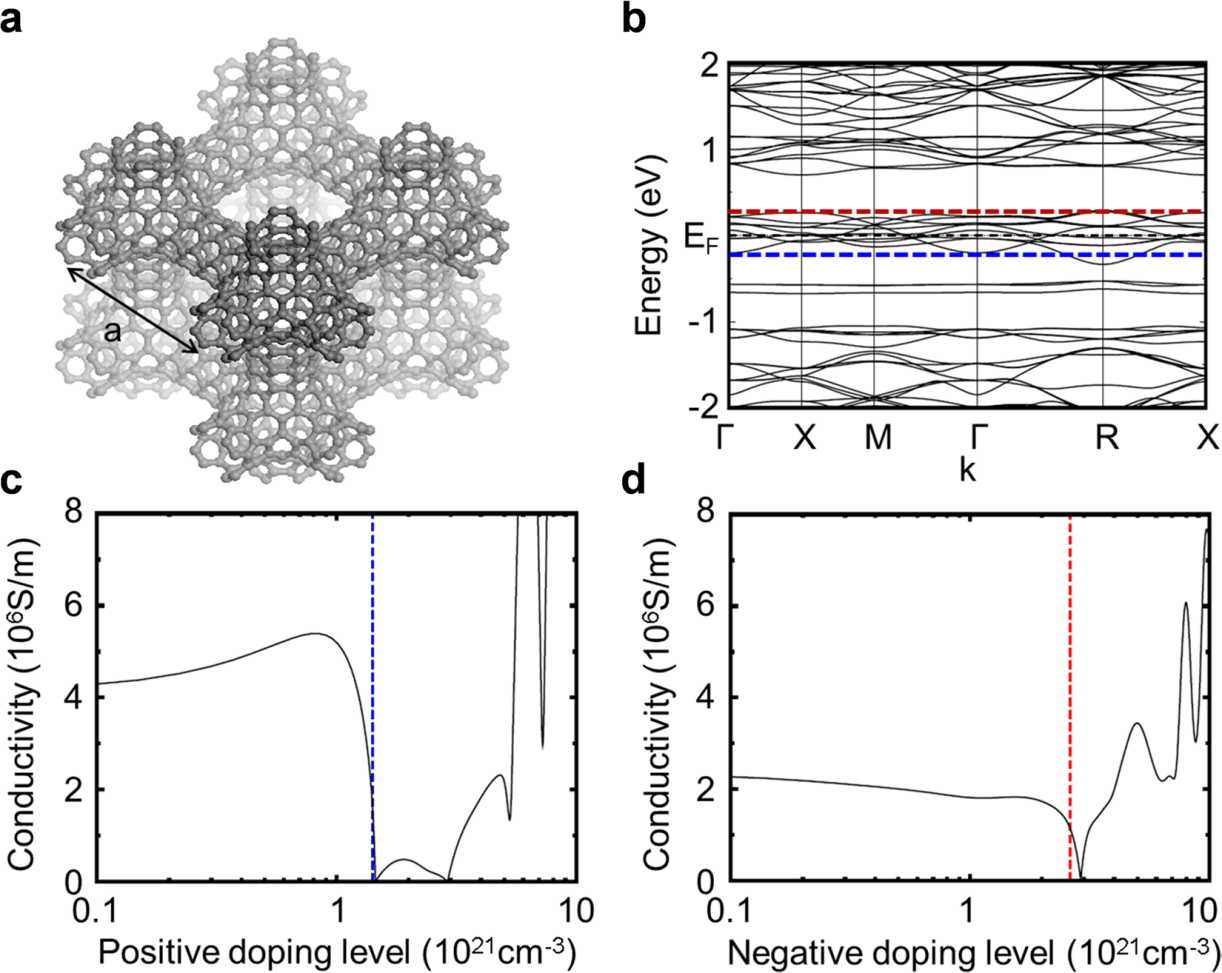
Electronic structure calculation. (**a**) Equilibrium geometry of a Schwarzite structure with 200 carbon atoms per cubic unit cell with a lattice constant *a*; model of the well-ordered pore 3D graphene-like zeolite-templated carbon framework. (**b**) Electronic band structure. The black dashed line represents the Fermi level of the electrically neutral system, while the red and blue dashed lines indicate the Fermi levels shifted by negative and positive doping, respectively, where the DP theory along with the effective mass approximation were applied. Evaluated electrical conductivities of the model system are shown as a function of (**c**) positive and (**d**) negative doping concentrations. The red and blue dashed vertical lines indicate the doping concentration corresponding to the energy levels marked with the same colored lines in (**b**).

To computationally evaluate the electrical conductivity of the well-ordered porous 3D graphene-like zeolite-templated carbon framework, we used a Schwarzite structure^28^ as the model shown in Fig. 5a. The unit cell contains a core with *O*_h_ symmetry and negative local Gaussian curvature; six (4,4) carbon nanotube segments connect the cores located in the neighboring unit cells. Our electronic structure calculation based on DFT revealed that the Schwarzite structure is metallic^28^, as shown in Fig. 5b. With the electronic band structure, its conductivity was calculated using the Boltzmann Transport (BT)^29^ and deformation potential (DP) theories^30^. The conductivity tensor ${\sigma }_{\alpha \beta }( {\mathcal E} )$ for a given energy ${\mathcal E}$ can be calculated using^29^

1 $${\sigma }_{\alpha \beta }( {\mathcal E} )=\frac{1}{N}{\sum }_{n,{\bf{k}}}{\sigma }_{\alpha \beta }(n,{\bf{k}})\frac{\delta ( {\mathcal E} - {\mathcal E} (n,{\bf{k}}))}{d {\mathcal E} }$$

where *N* is the number of **k**-points sampled, ${{\sum }^{}}_{n,{\bf{k}}}$ is the summation over the band index *n* in the momentum **k**-space, and *δ*(*x*) is Dirac delta function. The conductivity tensor for a given *n* and **k** can be obtained using ${\sigma }_{\alpha \beta }(n,\,{\bf{k}})={e}^{2}\tau (n,\,{\bf{k}})\,{v}_{\alpha }(n,\,{\bf{k}}){v}_{\beta }(n,\,{\bf{k}})$. Here, *τ* is the relaxation time and ${v}_{\alpha }$ is the *α*-component of the group velocity ${\bf{v}}(n,\,{\bf{k}})={{\nabla }}_{{\rm{k}}} {\mathcal E} (n,\,{\bf{k}})$, where ${\mathcal E} (n,\,k)$ is the electronic band energy shown in Fig. 5b, and ${{\nabla }}_{{\rm{k}}}$ is the gradient in the momentum space.

To determine the electric conductivity $\sigma$, it is important to calculate the relaxation time $\tau$, which is difficult. Usually, because of such difficulty, the BT theory is used under a constant relaxation time approximation to get $\sigma /\tau$ rather than $\sigma$. However, in this study, we calculated the relaxation time $\tau$ using the DP theory^30^ along with the effective mass approximation, which provides the following relation

2 $${\boldsymbol{\tau }}=\frac{{\boldsymbol{\mu }}{{\boldsymbol{m}}}^{\ast }}{{\boldsymbol{e}}}=\frac{{\bf{2}}\sqrt{{\bf{2}}{\boldsymbol{\pi }}}{\boldsymbol{C}}{\hslash }^{{\bf{4}}}}{{\bf{3}}{({{\boldsymbol{k}}}_{{\boldsymbol{B}}}{\boldsymbol{T}})}^{{\bf{3}}{\boldsymbol{/}}{\bf{2}}}{{\boldsymbol{E}}}_{{\rm{DP}}}{{\boldsymbol{m}}}^{\ast {\bf{3}}{\boldsymbol{/}}{\bf{2}}}}$$

where *μ* is the carrier mobility, m* is the effective mass at the band edge, and ***e*** is the electronic charge. The elastic constant was obtained using $C=[{\partial }^{2}{E}_{t}/\partial {({\rm{\Delta }}L/{L}_{0})}^{2}]/{V}_{0}$, that is, the second derivative of the total energy *E*_*t*_ of the system with respect to the strain ${\rm{\Delta }}L/{l}_{0}$ along the transport direction. Here, *l*_0_ is the equilibrium length and *V*_0_ is the cell volume. From DP, *E*_DP_ was determined using ${\rm{\Delta }} {\mathcal E} /({\rm{\Delta }}l/{l}_{0})$ or the ratio of the strain-induced energy level shifts ${\rm{\Delta }} {\mathcal E}$ at the band edges to the strain. ${\rm{\Delta }} {\mathcal E}$ was evaluated with respect to the 1 *s* core level obtained using an all-electron calculation. In this study, only one transport direction was considered because of the simple cubic symmetry of our model system. Note that the DP theory was originally developed for hole and electron carriers located at the valence band maximum and at the conduction band minimum, respectively, in semiconductors. To apply the theory to our metallic system, two doping situations were considered. In each doping case, the Fermi level is located at either of the energy levels at the band edges specified with a blue or red dashed line in Fig. 5b, which can be achieved using either a positive or negative doping process, respectively. The elastic constant *C*, the effective mass m*, and the DP value *E*_DP_ were estimated by fitting various data obtained by applying the DP theory, as shown in Supplementary Fig. 7 (See Supplementary Information). The relaxation time $\tau$ and the mobility *μ* were then evaluated using Eq. 2 with these estimated parameters.

Figure 5c,d shows the electrical conductivity of our model system (i.e. Schwarzite) as a function of the doping levels that we calculated with all the necessary quantities evaluated, as mentioned above. In the positive (negative) doping case that resulted in the Fermi level of the system shifting down (up), the conductivity was calculated to be $\sigma =2 \sim 3\times {10}^{6}\,{\rm{S}}\,{{\rm{m}}}^{-1}$ ($\sim 1\times {10}^{6}\,{\rm{S}}\,{{\rm{m}}}^{-1}$), as shown in Fig. 5c (Fig. 5d). To verify our method, we calculated the relaxation time $\tau$ of graphene and thus its electrical conductivity $\sigma$ using the same method. They were calculated to be $\tau = \sim 24\,{\rm{ps}}$, and $\sigma =5\times {10}^{7}\,{\rm{S}}\,{{\rm{m}}}^{-1}$, which are in good agreement with experimental results^31, 32^. Our calculated conductivity for the model system is about an order of magnitude smaller than that of graphene, which is reasonably acceptable because of the structural differences between the two. Interestingly, the conductivity of the model system is comparable to that of normal metals. Even more interestingly, it is several orders of magnitude larger than that of amorphous carbon ($\sigma = \sim 2\times {10}^{3}\,{\rm{S}}\,{{\rm{m}}}^{-1}$)^33^. These theoretical results agree well with our experimental observations, though there are deviations of conductivity from the multipath through the carbon chains. Additionally, we measured the sheet resistance Rs of the zeolite-carbon composites that were deposited using the drop casting method on a printed circuit board substrate with a stripe patterned metal electrode (interval distance of 1 mm) using a probe station. Relatively high Rs of 67.5 kΩ/sq and infinite (higher than 100 MΩ/sq) were measured on the carbon with and without heat treatment (at 650 °C), respectively. We found that it was difficult to form a uniform film when depositing zeolite-carbon composites because particle formation occurs, rather than the geometrically uniform thin film that forms after drop casting. In addition, we found that it was difficult to obtain the absolute sheet resistance for the powder samples because there are many factors that influence the resistance (e.g. high contact resistance, low density and uniformity, and packing density), which makes the reliable measurement of electrical conductance on carbon samples with a macroscopic four-probe configuration quite challenging. In the case of C-AFM, we can make a reliable electrical contact with carbon particles with the AFM tip. Therefore, we think that this study shows a unique capability for nanoscale electrical measurements on carbon particles with C-AFM.

In conclusion, we demonstrate that the electrical conductance of LaY-templated carbon depends on the heat treatment of the carbon structure, which increases the C–C bonds within the narrow necks between neighboring supercages. The higher conductance of the LaY-templated carbon with heat treatment than that of the LaY-templated carbon without heat treatment or low synthesis temperature is revealed because of the loss of pore order and differing hydrogen content. The crystallinity of these microporous carbon products is confirmed using HR-TEM and XRD measurements, and the results are well matched with the conductance trends. In addition, the electrical conductivity of the LaY-templated carbon was computed using the Boltzmann transport theory and the deformation potential theory based on density functional theory. These results suggest that the degree of order for the pores in the 3D ZTC structures is related to the electrical conductivity and the LaY-templated carbon is potentially applicable in supercapacitors and rechargeable batteries.

## Methods

### Synthesis of LaY-templated carbon

Y zeolite was synthesized according to methods found in the literature^34, 35^. Ion exchange was performed with a 0.5 M aqueous solution of lanthanum nitrate hexahydrate (98%, Sigma-Aldrich) at room temperature. For carbon deposition within the La^3+^ form of the Y zeolite (i.e. LaY zeolite), 0.5 g of LaY zeolite was placed in a quartz plug-flow reactor equipped with a 15-mm diameter fritted disk. The reactor was then heated to different temperatures (i.e. 500 °C and 650 °C) with a heating rate of 5 °C min^−1^ under dry N_2_ flow. After stabilizing the temperature at the desired value, a mixture of ethylene, N_2_, and water vapor (10/87/3) was passed through the sample bed. The flow rate of the gas mixture was 130 cm^3^ min^−1^. Injection of the ethylene gas and water vapor was stopped when carbon deposition was complete. The carbon deposition process gave a carbon yield of approximately 0.34 g per 1 g zeolite, which was confirmed using thermogravimetric analysis. After carbon deposition, the reactor was heated to 900 °C under N_2_ flow, and this temperature was maintained for 2 h to rigidify the carbon frameworks formed in the LaY zeolite. After cooling the reactor, the resultant sample was repeatedly treated in a 0.3 M HF/0.15 M HCl solution to remove the LaY zeolite. The template-free carbon products were collected using filtration, washed with deionized water, and dried at 100 °C. Complete removal of the zeolite template was confirmed by thermogravimetry using a TGA Q50 (TA Instruments) (Supplementary Fig. 8). The final carbon samples were denoted as C500-HT and C650-HT, according to the carbon deposition temperature. For comparison, a carbon sample was also synthesized through carbon deposition at 650 °C without the subsequent thermal treatment at 900 °C. The carbon product, thus obtained, is denoted as C650. The synthesized LaY-templated carbon was dispersed in isopropanol for drop casting on a Au (111) substrate. After drop casting, the LaY-templated carbon was randomly dispersed and stacked on the Au (111) substrate. The Au (111) film was prepared using flame annealing after e-beam evaporation of a 100-nm thick Au layer on a mica substrate.

### Characterization of LaY-templated carbon

Characterization of the LaY-templated carbon was performed using high-resolution transmission electron microscopy (HR-TEM), XRD, SEM, and XPS. The crystallinity of the LaY-templated carbon was determined using HR-TEM (FEI Titan E-TEM G2) with 300 kV acceleration using a holey carbon grid (300 mesh) after ethanol dispersion on the support. SEM images were obtained using a Magellan 400 (FEI company) at a bias voltage of 2 kV and work distance of 4.1 mm. XPS data for the LaY-templated carbon on Au (111) were taken using a Thermo VG Scientific Sigma Probe spectrometer equipped with an Al-Kα X-ray source (1486.3 eV). Powder XRD patterns were recorded on a Rigaku Multiplex instrument using Cu Kα radiation (30 kV, 40 mA).

### AFM measurements

The electrical conductance and morphology of the LaY-templated carbon on Au (111) substrate were characterized using an environmental AFM (5500, Agilent) in combined conductance sensing mode with a Pt/Ir-coated AFM probe (PPP-EFM-50, Nanosensors) in air. A crushed ZTC sample was measured using C-AFM. The AFM tip has a typical force constant of 2.8 N/m and resonance frequency of 75 kHz. The topographical images were taken using non-contact mode AFM, then the I–V curves of the local positions were obtained in I–V spectroscopy mode. We confirm that the tip–sample contact is in the elastic regime by using a series of reproducible measurements of the I–V curves that indicate the absence of plastic deformation of the tip and the sample.

### Theoretical calculations

A first-principles calculation based on DFT was performed using the DMol^3^ package^36^ to study the electronic structures and electrical conductance of the schwarzite structure^28^ selected as the model for the well-ordered pore 3D graphene-like zeolite-templated carbon framework. We used the generalized gradient approximation with the Perdew–Burke–Ernzerhof ^37^ functional to describe the exchange-correlations functional. A double numerical basis set was chosen with a real-space cutoff of 3.7 Å to expand the electronic wave functions in the all-electron calculation. The octupole scheme for the multipolar fitting procedure and a fine grid scheme for numerical integration were employed to accurately evaluate the charge density. We performed structure relaxation using the Broyden–Fletcher–Goldfarb–Shanno algorithm^38–43^ without any symmetry constraints until all the atomic forces became smaller than 4 × 10^−4^ Ha Å^−1^. For such structural relaxation, only the Г point was used for the Brillouin zone integration since the model schwarzite structure is very large. The electronic transport properties were calculated using the semi-classical BT theory^29^ with the relaxation time obtained using the DP theory^30^. To evaluate reliable transport coefficients, a denser 2 × 2 × 2 *k*-grid was used for the Monkhorst–Pack scheme^44^.

## Electronic supplementary material

Supplementary information
