# Supplementary material for: Extremely high electrical conductance of microporous 3D graphene-like zeolite-templated carbon framework
Source: Sci Rep. 2017 Sep 13;7:11460. doi: 10.1038/s41598-017-11602-5 (PMC5597609; doi:10.1038/s41598-017-11602-5)
Supplement: Supplementary file 1 — Supplementary information [file 41598_2017_11602_MOESM1_ESM.doc]

**SUPPLEMENTARY INFORMATION**

**Extremely high electrical conductance of microporous 3D graphene-like zeolite-templated carbon framework**

**Hyunsoo Lee,^1†^ Kyoungsoo Kim,^1,6†^ Seoung-Hun Kang,^2,3^ Yonghyun Kwon,^1,4^ Jong Hoon Kim,^1^ Young-Kyun Kwon, ^2,3^ Ryong Ryoo,^1,4^ and Jeong Young Park,^1,5^**

^1^Center for Nanomaterials and Chemical Reactions, Institute for Basic Science (IBS), Daejeon 34141, South Korea.

^2^Department of Physics and Research Institute for Basic Sciences, Kyung Hee University, Seoul, 02447, South Korea.

^3^Korea Institute for Advanced Study, Seoul 02455, South Korea.

^4^Department of Chemistry, Korea Advanced Institute of Science and Technology (KAIST), Daejeon 34141, South Korea.

^5^Graduate School of EEWS, Korea Advanced Institute of Science and Technology (KAIST), Daejeon 34141, South Korea.

^6^Current address: Department of Chemistry, Chonbuk National University, Jeonju, Jeollabuk-do, 54896, South Korea.

† Authors contributed equally to this work. Correspondence and requests for materials should be addressed to Y.-K.K. (email: ykkwon@khu.ac.kr) or to R.R. (email: rryoo@kaist.ac.kr) or to J.Y.P. (email: jeongypark@kaist.ac.kr).

1. **NMR spectra and elemental analysis of carbon samples**


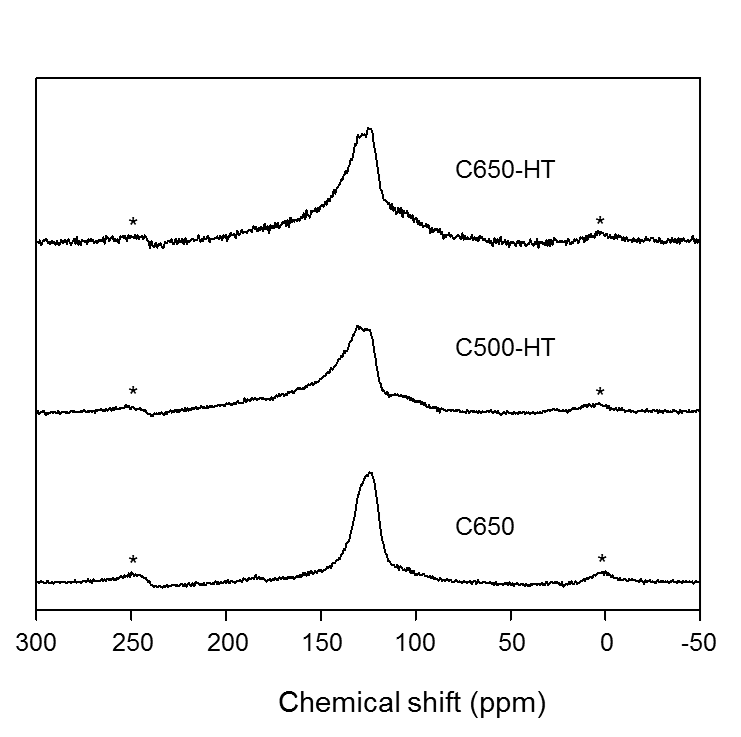


Supplementary Figure 1. Magic-angle spinning solid-state ^13^C NMR spectra of carbon samples obtained using LaY zeolite as the template. The peak at 123 ppm can be assigned to a six-membered ring of *sp*^2^ carbon; the peak at 129 ppm can be attributed to a five- or seven-membered ring that has smaller C–C–C angles in the conjugated *sp*^2^ carbon system. The negligible peak at 183 ppm can be attributed to oxygen functional groups.

**Supplementary Table 1.** Elemental composition of the carbon samples


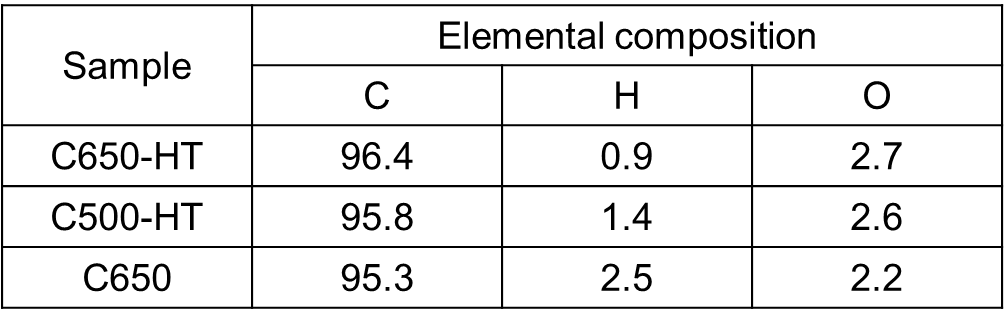


1. **Raman spectra of carbon samples**

**Supplementary Figure 2.** Raman spectra of C500-HT, C650-HT, and C650 samples.

1. **Scanning electron microscopy (SEM) images**

**Supplementary Figure 3.** SEM images at (left) 10000
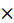
 and (right) 50000
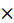
 magnification of the LaY-templated carbon on a Au (111) substrate for (a) C650-HT and (b) C650.

1. **Morphology of carbon before and after I–V measurements**

**Supplementary Figure 4.** Topographical images (2.6
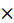
 10.0 µm^2^) of C650 on Au (111) (a) before and (b) after I–V measurements. The yellow dotted circles show the I–V curve measured on C650 in Figure 3.

1. **Conductive-AFM results of the LaY-templated carbon synthesized at low temperature (C500-HT)**

**Supplementary Figure 5.** (a) Topography (1.5
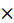
 1.5 µm^2^) of C500-HT on Au (111). (b) Height line profile along the solid red line in (a). (c) I–V curves measured on C500-HT and Au (111) with a tip sweep bias of
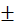
1 V in air. (d) Local electrical conductance of C500-HT and the Au (111) surface measured in (a). The white dotted arrow with numbers in (a) indicates the direction and sequence of the I–V measurements.

1. **X-ray photoelectron spectroscopy (XPS) measurements**

**Supplementary Figure 6.** XPS spectra: C 1s spectra of the LaY-templated carbon on (a) C650-HT, (b) C650, and (c) C500-HT. O 1s spectra of the LaY-templated carbon on (d) C650-HT, (e) C650, and (f) C500-HT. The table shows the portion (%) of each group in the C 1s spectra.

1. **Elastic constant, effective mass, and deformation potential energy**


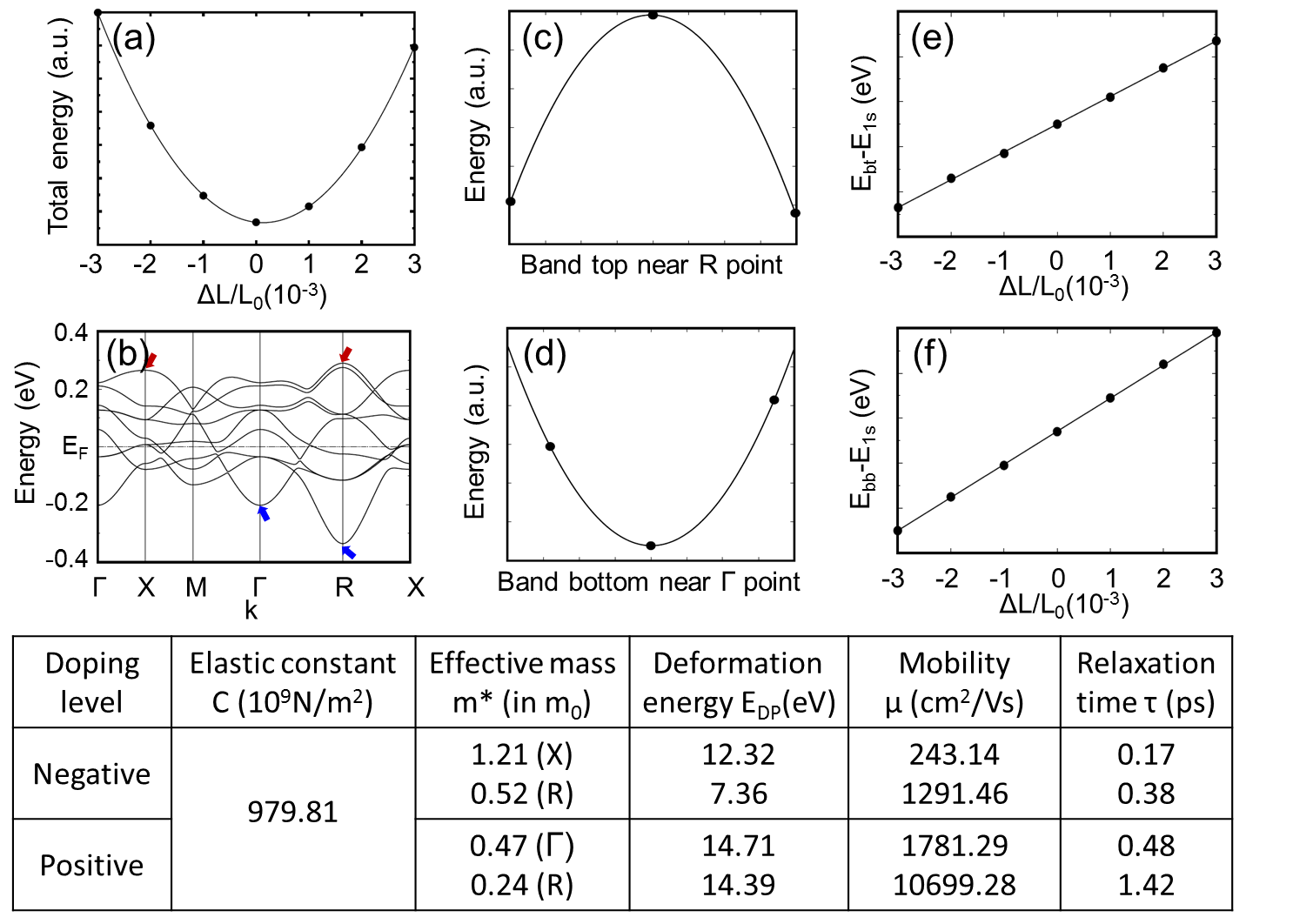


**Supplementary Figure 7.** (a) Calculated total energy of the schwarzite structure, a model of the well-ordered pore 3D graphene-like zeolite-templated carbon framework as a function of strain. The elastic constant C was obtained by taking the second derivative of the fitting function of the total with respect to the strain
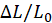
 along the transport direction. (b) Band structure near the Fermi level
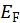
. The red and blue arrows indicate band edges to which the Fermi level can be shifted by negative and positive doping processes. DP theory and effective mass approximation were applied to these edge levels. For instance, (c) and (d) show the band edge top near the R point and the band edge bottom near the Г point. These bands were fitted to harmonic functions to evaluate their corresponding effective masses. (e) and (f) display the strain
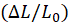
-induced energy level shifts
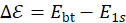
 and
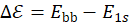
, where
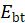
,
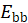
, and
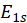
 are the band edge top, bottom, and 1*s* core level energies, respectively. The DP energy values
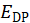
 were obtained from the slopes of their linear fitting functions. The table shows not only the evaluated values of the three quantities mentioned above, but also the values of the mobility
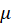
 and relaxation time
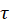
 obtained using eq 1 in the main text.

1. **Thermogravimetric analysis of carbon samples**

**Supplementary Figure 8.** Weight loss curves of carbon samples measured using thermogravimetric analysis under air flow. The curves exhibit a negligible amount of sample residue after heating to 800 °C.
